# Unsaturated Fatty Acid Intake During Periconception and Incidence of Achieving Pregnancy: A Systematic Review and Meta-Analysis

**DOI:** 10.3389/fphys.2021.757266

**Published:** 2021-11-15

**Authors:** Cintia Romina Gatti, Dalmiro Gomez Ribot, Javier Mariani, Alicia Jawerbaum

**Affiliations:** ^1^Facultad de Medicina, Universidad de Buenos Aires, Buenos Aires, Argentina; ^2^Laboratory of Reproduction and Metabolism, Concejo Nacional de Investigaciones Científicas y Técnicas (CONICET)-Universidad de Buenos Aires, Centro de Estudios Farmacológicos y Botánicos (CEFYBO), Buenos Aires, Argentina; ^3^Hospital de Alta Complejidad “El Cruce”, Florencio Varela, Buenos Aires, Argentina

**Keywords:** periconceptional period, MUFA, PUFA, maternal diet, pregnancy success

## Abstract

**Background:** Previous studies suggest that maternal diets enriched in unsaturated fatty acids may have a positive effect on pregnancy success. The aim of the present study was to conduct a systematic review and meta-analysis to evaluate whether increased dietary intake of polyunsaturated fatty acids (PUFAs) or monounsaturated fatty acids (MUFAs) during the periconception period has beneficial effects on the achievement of pregnancy in women.

**Methods:** The electronic databases PubMed, Medline and Cochrane Central Register, as well as references in related review articles, were searched to find studies assessing the effects of unsaturated fatty acid dietary intake during the periconception period on the achievement of pregnancy in women. Pregnancy was confirmed by high levels of hCG (biochemical means) and ultrasound confirmation of a gestational sac and heartbeat (clinical means).

**Results:** For the meta-analysis evaluating the effects of periconceptional diets enriched in PUFAs on pregnancy, four articles, providing data on 2,121 patients, were included. Results showed that periconceptional intake of PUFAs has no significant effects on achieving pregnancy compared to controls, according to both the fixed effects and random effects models (RR = 0.99, 95% CI 0.98–1.00). Further secondary analysis considering ω-6 and ω-3 PUFAs separately showed no significant effects on achieving pregnancy compared to controls. On the other hand, for the meta-analysis evaluating the effects of periconceptional diets enriched in MUFAs on achieving pregnancy, five articles, providing data on 2,473 patients, were included. Results showed that periconceptional dietary intake of MUFAs has significant effects on achieving pregnancy compared to controls according to the fixed effects model (RR = 1.03, 95% CI 1.01–1.06, *p* < 0.02) but not to the random effects model, due to heterogeneity. A secondary meta-analysis excluding one study which led to heterogeneity showed significant effects of MUFAs on achieving pregnancy compared to controls, according to both the fixed effects and random effects models (RR = 1.03, 95% CI 1.01–1.05, *p* < 0.02).

**Conclusion:** The meta-analysis of published clinical studies suggests that diets enriched in MUFAs, although not those enriched in PUFAs, may have a positive effect on pregnancy success as determined by HCG and ultrasonography.

**Systematic Review Registration:** https://www.crd.york.ac.uk/PROSPERO/display_record.php?ID=CRD42021239355, identifier: CRD42021239355.

## Introduction

The complex processes of decidualization, ovulation and implantation, which are important periconceptional determinants of maternal, placental and fetal health, as well as of the child's health later in life, can be modulated by nutrigenomic agents (Norwitz et al., 2001; Sturmey et al., 2009; Gaskins and Chavarro, 2018; Ng et al., 2020). Unsaturated fatty acids are relevant nutrigenomic agents, capable of regulating nuclear receptors such as peroxisome proliferator activated receptors (PPARs) and to exert potent antioxidant and anti-inflammatory effects both dependently and independently of PPAR activation (Jawerbaum and Capobianco, 2011; Georgiadi and Kersten, 2012; Bordoni et al., 2021). Unsaturated fatty acids include monounsaturated fatty acids (MUFAs) and polyunsaturated fatty acids (PUFAs).

The most common dietary source of MUFAs is olive oil, which is highly enriched in oleic acid (18:1 ω-9). In particular, extra virgin olive oil is the base of the Mediterranean diet (Wongwarawipat et al., 2018), a diet highly recognized for its capacity to prevent cardiovascular diseases and to exert potent antioxidant effects (Estruch et al., 2018; Wongwarawipat et al., 2018). PUFAs include fatty acids of the ω-3 and ω-6 series. The precursors of each series have to be obtained through the diet, as they cannot be synthesized by human enzymes (Bordoni et al., 2021). PUFAs of the ω-6 series such as arachidonic acid (20:4) are mostly contained in meat, whereas others such as linoleic acid (18:2) are mainly found in plants, highly concentrated in seeds and vegetable oils like sunflower, safflower and canola oils. On the other hand, PUFAs of the ω-3 series such as eicosapentanoic acid (EPA, 20:5) and docosahexaenoic acid (DHA, 22:6) are mostly found in fish, seafood and algae, while others such as alpha-linolenic acid (ALA, 18:3) are mostly found in plants, highly concentrated in seeds and vegetable oils such as canola and chia oils (Bordoni et al., 2021). PUFAs are highly relevant in reproductive processes (Herrera, 2002; Wathes et al., 2007). Indeed, PUFAs of the ω-6 and ω-3 pathways are essential for pregnancy success, and highly involved in the appropriate timing of the anti-inflammatory and pro-inflammatory reactions needed for the physiological processes of ovulation, decidualization and implantation (Norwitz et al., 2001; Jawerbaum and Gonzalez, 2005).

By using experimental models of diabetes and pregnancy, characterized by a pro-oxidant and pro-inflammatory intrauterine environment, we have previously found that dietary supplementation with either MUFAs or PUFAs during pregnancy prevents resorption and malformation rates (Higa et al., 2010). Moreover, dietary supplementation with MUFAs, ω-6 PUFAs and ω-3 PUFAs improves rat decidualization as well as PPAR and mechanistic target of rapamycin (mTOR) signaling pathways, relevant for the histotrophic nutrition of the embryo (Higa et al., 2014; Roberti et al., 2021). In addition, the interaction of mTOR and PPAR systems, two systems regulated by nutrients, has been found to be critical for decidualization and pregnancy success (Roberti et al., 2018).

In women, different periconceptional dietary guidelines include recommendations on optimal intakes of MUFAs, ω-6 PUFAs and ω-3 PUFAs (Hanson et al., 2015; Sioen et al., 2017). Nevertheless, different studies in women have shown that unsaturated fatty acid intake at preconception stages or during pregnancy is less than optimal (Sioen et al., 2017).

Several studies have suggested that supplementation or increased intake of different kinds of unsaturated fatty acids may benefit fertility, although others have shown no effect (Gaskins and Chavarro, 2018; Lass and Belluzzi, 2019). To our knowledge, no previous systematic reviews or meta-analyses addressing this issue have been reported. Based on this, we here conducted a systematic review and meta-analysis to evaluate whether unsaturated fatty acid supplementation during the periconception period has beneficial effects on the achievement of pregnancy in women.

## Materials and Methods

The protocol for our study is registered in the international prospective register of systematic reviews (PROSPERO). Registration number CRD42021239355 (available from https://www.crd.york.ac.uk/PROSPERO/display_record.php?ID=CRD42021239355). This systemic review was conducted following the PRISMA method and recommendations (Moher et al., 2009).

### Search Methods

We searched the electronic databases PubMed, Medline and Cochrane Central Register to identify studies assessing the effects of unsaturated fatty acid dietary intake during the periconception period on the achievement of pregnancy in women. The last search was performed in May 2021, and there was no imposed restriction regarding country or ethnicity. We also searched the references in field-related review articles from January 2019 to May 2021. Databases were searched using the following PubMed's MeSH terms, which were selected with a topic-specific strategy: (Polyunsaturated fatty acid OR monounsaturated fatty acid OR Mediterranean diet OR MUFA OR PUFA OR olive oil OR omega-3 fatty acid OR omega-6 fatty acid OR dietary fat intake OR n-3 fatty acid OR n-6 fatty acid) AND (preconception OR periconception OR assisted reproduction OR IVF) AND (pregnancy OR fecundability OR fertility).

### Selection of Studies

The population evaluated were women who were attempting to become pregnant. We included randomized controlled trials, clinical trials and prospective cohort studies that evaluated: (i) the supplementation (intervention group) of PUFAs or MUFAs compared with no supplementation (placebo or control groups), (ii) an increased dietary intake pattern of PUFAs or MUFAs (intervention group) compared with a low intake pattern of PUFAs or MUFAs (control group), and (iii) a dietary intake which leads to increased circulating PUFAs or MUFAs (intervention group) compared to low circulating PUFAs or MUFAs (control group). There were no restrictions based on dosage, administration, or consumption forms and no restrictions based on maternal age. All studies had to report data on pregnancy (diagnosed either clinically or biochemically) as relative risks (RRs) with 95% confidence interval (95% CI) values or other values which could be converted into RRs. Adjustments for potential confounders were conducted at study level, as thus incorporated into the analyses by using adjusted estimates from primary studies.

### Data Extraction and Management

The primary outcome measure of this review was the incidence of pregnancy. Pregnancy achievement was defined with a positive clinical and/or biochemical diagnosis: an ultrasound confirmation of a gestational sac and heartbeat (clinical diagnosis) and/or high levels of hCG (biochemical diagnosis). Two of the authors (CR-G and D-GR) independently extracted and tabulated data from each study, by using a standard extraction form. Discordances were solved by A-J, an experienced senior researcher. The information extracted included: the first author's name, year of publication, study design, number of participants and groups, timing and length of interventions, type of interventions and their evaluation, participant characteristics, main outcome (pregnancy diagnosed by clinical/biochemical methods) and main conclusions of each article. Studies included in this meta-analysis varied in terms of the methods, timing, and type of intervention. Seven studies addressing dietary intake/circulating levels of the different types of unsaturated fatty acids (MUFAs, PUFAs, ω-3 PUFAs, and/or ω-6 PUFAs) were explored.

### Statistical Analysis

We used RRs with 95% CIs to test the relationship between unsaturated fatty acids and the achievement of pregnancy. Study-specific RRs were pooled using the inverse variance method with the metagen function and evaluated by the RStudio version 1.3.1093 Software for Windows. Both fixed and random effects models were performed and analyzed. These models differently compute the variance around the effect estimates, being the random effects model the one that estimates the variability between studies. The covariate adjustment was considered in the step prior to pooling, since adjusted estimates were used. Heterogeneity was evaluated by the *I*^2^ statistic and Cochran's Q-test, and an *I*^2^ higher than 50% or a *P*-value of the Q-test lower than 0.10 was considered significant heterogeneity. Pooled estimates show the *p*-value for overall effects (i.e., the type I error of the test). Confidence intervals were considered as indicative of significance in cases of exclusion of the null (i.e., OR 1). Funnel plots were constructed to assess publication bias by visual inspection, as tests for asymmetry were not conducted due to the small number of studies.

## Results

### Study Selection

**Figure 1:**
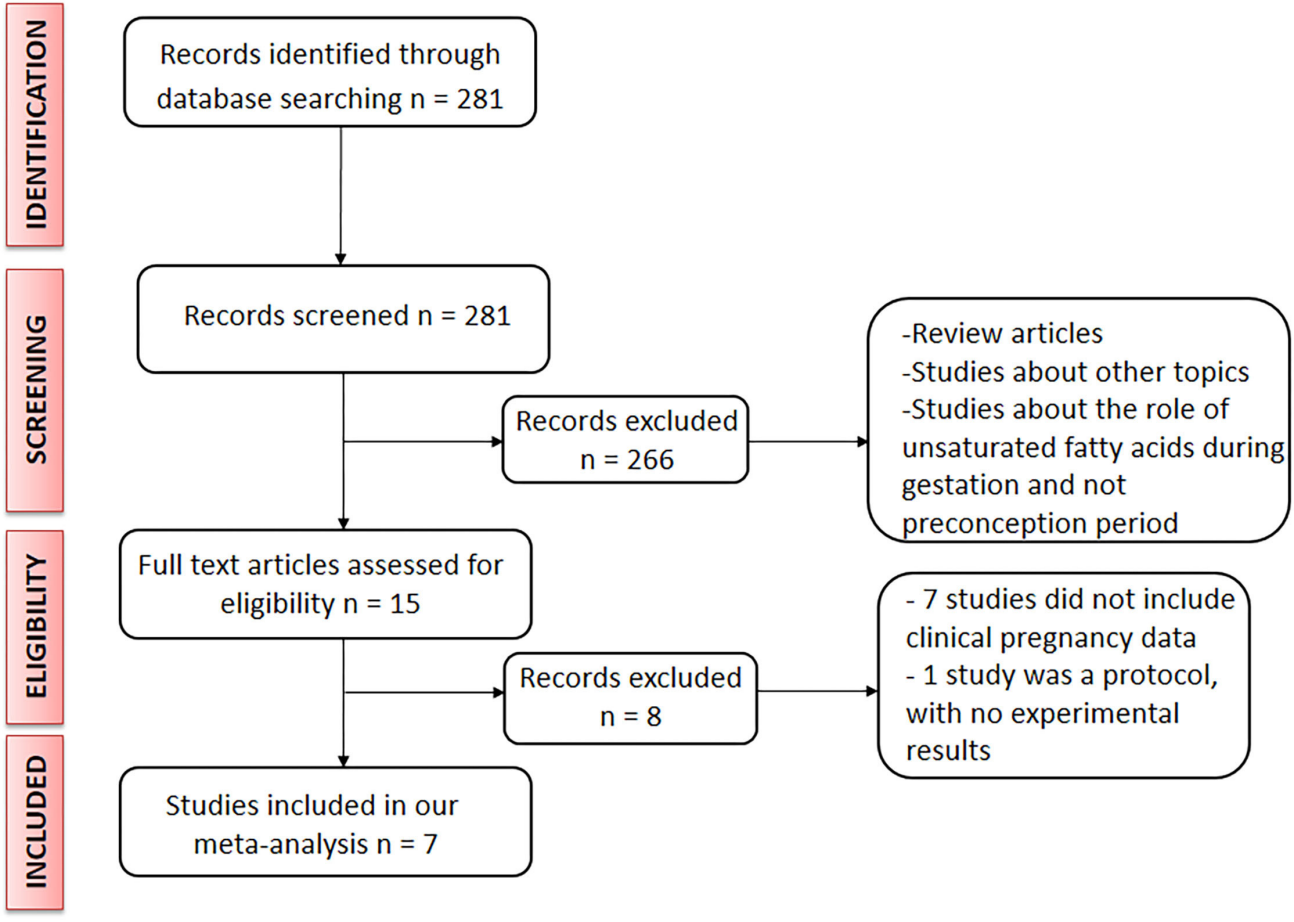
Flow diagram of selection of studies eligible for our meta-analysis.

The literature search procedure is presented in Figure 1. We found 281 articles, 15 of which met the inclusion criteria and were selected as appropriate for inclusion in this meta-analysis. After evaluation of the title, abstract, type of article and/or full text, 266 articles were excluded. The main reasons for exclusion were: main purpose not related to the content of the present study, evaluation of the role of unsaturated fatty acids during pregnancy but not during the periconception period, and review articles. After carefully addressing the full text of the 15 articles selected, eight more were excluded as they lacked the data needed for this evaluation (data on clinical/biochemical diagnosis of pregnancy achievement and/or appropriate data to be used to evaluate the periconceptional unsaturated fatty acid intake). Thus, only seven articles were finally included in our meta-analysis.

The study design characteristics of the seven studies identified by database searches and secondary referencing that were included in this meta-analysis are shown in Table 1. Four of them were eligible to evaluate the association between PUFA dietary intake and achieving pregnancy, while five of them were eligible to evaluate the association between MUFA dietary intake and achieving pregnancy. These five studies included three studies addressing the effect of a Mediterranean diet, highly enriched in MUFAs. Fatty acid intake was reported as percent of total energy in one work, as grams per day in one work and as a score reflecting fatty acid intake in three works. Fatty acid concentrations in plasma, expressed as percent of total fats, were reported in two works.

**Table 1:** Characteristis of studies on the association between periconceptional intake of unsaturated fatty acids and pregnancy.

**References**	**Study design**	**Groups (*n*)**	**Follow up**	**Intervention**	**Evaluation of intervention**	**Participants**	**Main conclusions**
Wise et al. (2018)	Prospective Cohort Study	Pregnancy Study Online (PRESTO) in North America. Control (358) Intervention (346) Snart Foraeldre (SF) in Denmark. Control (283) Intervention (286)	12 months	Dietary fat intake was classified into quartiles according to the data distribution of the energy percentage of each type of fat (MUFAs, PUFAs, ω-3 and ω-6 PUFAs) in the diet. The fourth quartile (upper quartile for each unsaturated fatty acid) was considered the intervention group. The first quartile (lower quartile for each unsaturated fatty acid) was considered the control group	Food Frequency Questionnaire (FFQ). Total dietary fat intake was calculated by summing all servings of fat from individual foods and mixed recipes	Women age 21–45 years and notreceiving fertility treatment Women age 18–45 years and not receiving fertility treatment	Pregnant women showed little evidence of an association between fecundability and intakes of total dietary fat, MUFAs, PUFAs, and ω-6 PUFAs. Low intake of ω-6 PUFAs was associated with reduced fecundity in the North America group
Mumford et al. (2018)	Prospective Cohort Study	Control (356) Intervention (356)	6 years	According to fatty acid measurements (MUFAs, PUFAs, and ω-3 PUFAs and ω-6 PUFAs), patients were classified into tertiles. The third tertile (highest plasma level of fatty acids) was considered the intervention group. The first tertile (lower plasma levels of fatty acids) was considered the control group	Plasma fatty acid analysis	Women age 18–40 years. BMI < 25 kg/ m^2^	Plasma fatty acid composition was associated with fecundability among normal-weight women
Chiu et al. (2018)	Prospective Cohort Study	Intervention (33) Control (33)	1 year	According to fatty acid measurements (total PUFAs, ω-3 and ω-6 PUFAs), patients were classified into tertiles. The third tertile (highest plasma level of fatty acids) was considered the intervention Group. The first tertile (lower plasma levels of fatty acids) was considered the control group	Plasma fatty acid analysis	Women included underwent 136 ART cycles within 1 year of blood sample collection. BMI < 25 kg/ m^2^	Higher serum levels of ω-3 PUFAs was positively related to the probability of clinical pregnancy and live birth, among women undergoing ART
Kermack et al. (2020)	Double blind randomized controlled trial	Control (56) Intervention (55)	6 weeks	Intervention group received olive oil for cooking, an olive oil– based spread, and a daily supplement drink enriched with EPA (800 mg), DHA (1,200 mg), and vitamin D (10 mg). Control group received sunflower seed oil for cooking, a sunflower seed oil–based spread, and a daily supplement drink without EPA, DHA, or vitamin D	Food Frequency Questionnaire (FFQ) and plasma fatty acid analysis	Women age 18–41 years. BMI 18–32 kg/m^2^	Pre-conceptional nutritional status can affect embryo development
Karayiannis et al. (2018)	Prospective Cohort Study	Control (79) Intervention (86)	6 months	Mediterranean diet intake was classified into tertiles according to the consumption of each type of food in the diet, including oils. The third tertile (upper adherence to Mediterranean diet) was considered the intervention group. The first tertile (lowest adherence to Mediterranean diet) was considered the control group	To evaluate the level of adherence to the Mediterranean diet, the MedDietScore was calculated for each participant considering the consumption of food items from nine food groups, including olive oil as a main component of this diet	Non-obese women 22–41 years of age, BMI < 30 kg/m^2^ who underwent a first IVF treatment	A higher adherence to the Mediterranean diet is associated with increased chance of clinical pregnancy and live birth after IVF/ICSI treatment in non-obese women, < 35 years of age
Ricci et al. (2019)	Prospective Cohort Study	Control (132) Intervention (142)	2 weeks	Mediterranean diet intake was classified into tertiles according to the consumption of each type of food in the diet, including oils. The third tertile (upper adherence to Mediterranean diet) was considered the intervention group. The first tertile (lower adherence to Mediterranean diet) was considered the control group	MedDietScore and Food Frequency Questionnaire (FFQ)	Women age 27–45 years. BMI = 22.3 kg/m^2^ (twenty nine women were obese (BMI >30 kg/ m^2^)	No linear association between adherence to a Mediterranean diet and oocyte quality or successful IVF
Vujkovic et al. (2010)	Observational prospective study	Control (54) Intervention (53)	16 months	Mediterranean diet intake was classified into tertiles according to the consumption of each type of food in the diet, including oils. The third tertile (upper adherence to Mediterranean diet) was considered the intervention group. The first tertile (lower adherence to Mediterranean diet) was considered the control group	MedDietScore. Food frequency questionnaire (FFQ).	Subfertile couples undergoing IVF/ICSI treatment	A high adherence to the Mediterranean diet by the couple may improve the chance of pregnancy after IVF/ICSI treatment

*MUFAs, monounsaturated fatty acids; PUFAs, polyunsaturated fatty acids; ω-3 PUFA, omega-3 polyunsaturated fatty acid; ω-6 PUFA, omega-6 polyunsaturated fatty acid; FFQ, Food Frequency Questionnaire; BMI, body mass index; ART, assisted reproduction treatment; EPA, eicosapentaenoic acid; DHA, docosahexaenoic acid; IVF, in vitro fertilization; ICSI, intracytoplasmic sperm injection*.

### Study Characteristics

The characteristics of the seven studies eligible to address the association between PUFA or MUFA dietary intake and the achievement of pregnancy are shown in Table 1. One of these seven studies reported results on two different populations that were separately addressed and thus separately included in the meta-analysis (Wise et al., 2018). Three of the studies were eligible to address the association between ω-6 PUFA intake and the achievement of pregnancy and four to evaluate the association between ω-3 PUFA intake and the achievement of pregnancy.

The eligible seven studies were published from 2010 to 2019, the total number of recruited participants was 2,708, the number of recruited participants in the different studies ranged from 33 to 356, and their age ranged from 18 to 45 years old. Four studies provided adjusted RRs, three studies provided adjusted ORs and one study provided values which could be converted into RRs.

### PUFAs and Pregnancy

**Figure 2:**
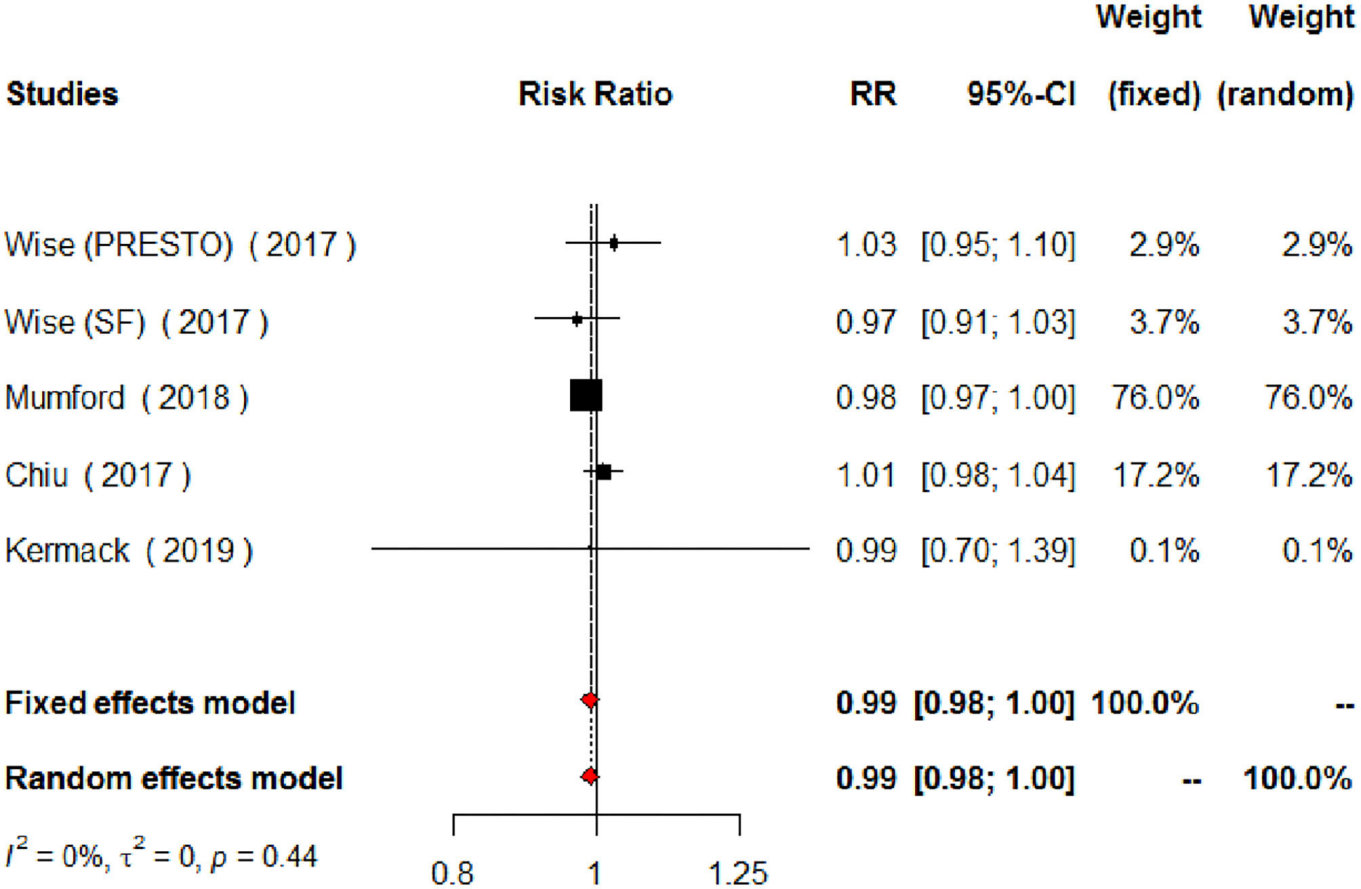
Forest plot for the meta-analysis of the relationship between periconceptional intake of PUFAs and pregnancy achievement (*n* = 2,121).

**Figure 3:**
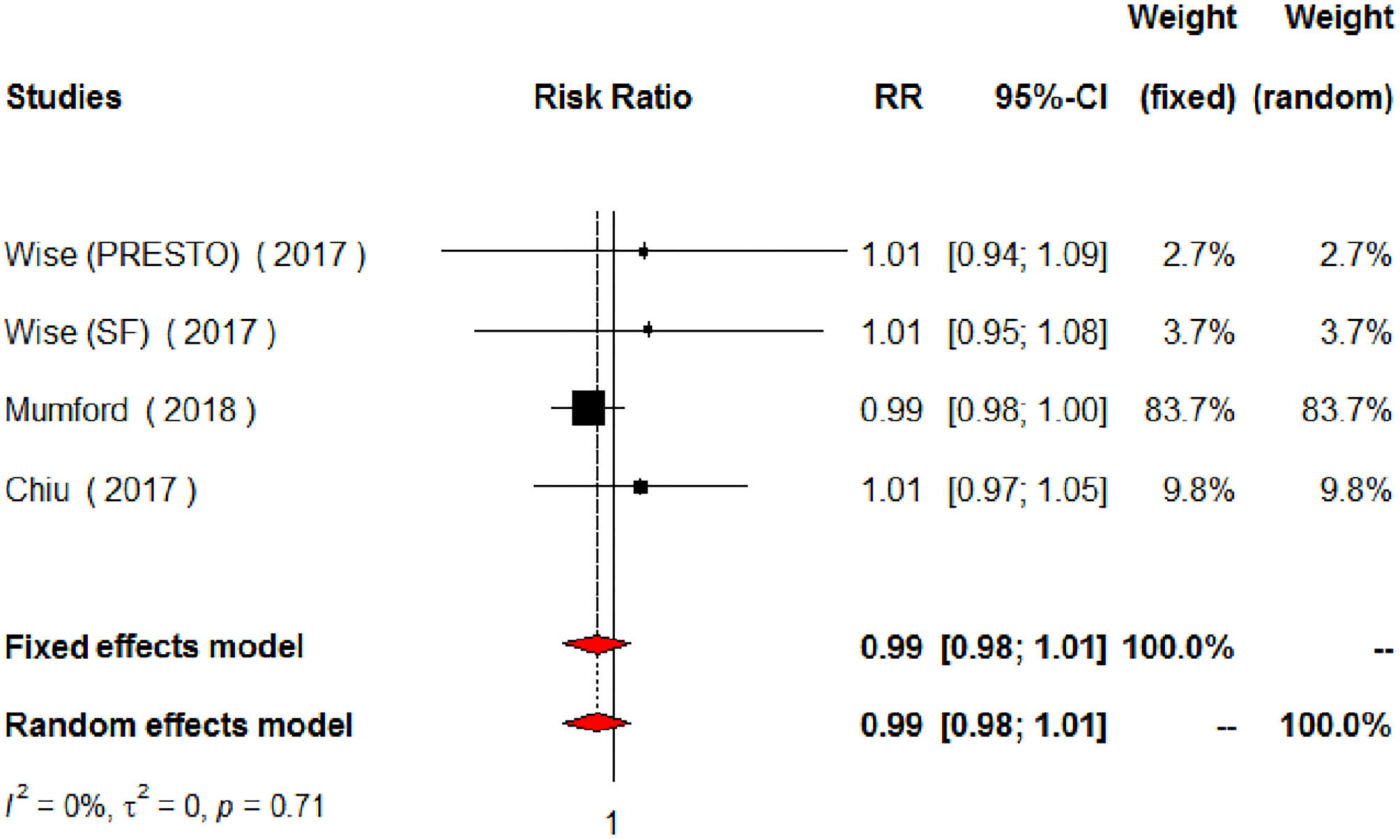
Forest plot for the meta-analysis of the relationship between periconceptional intake of ω-6 PUFAs and pregnancy achievement (*n* = 2,002).

**Figure 4:**
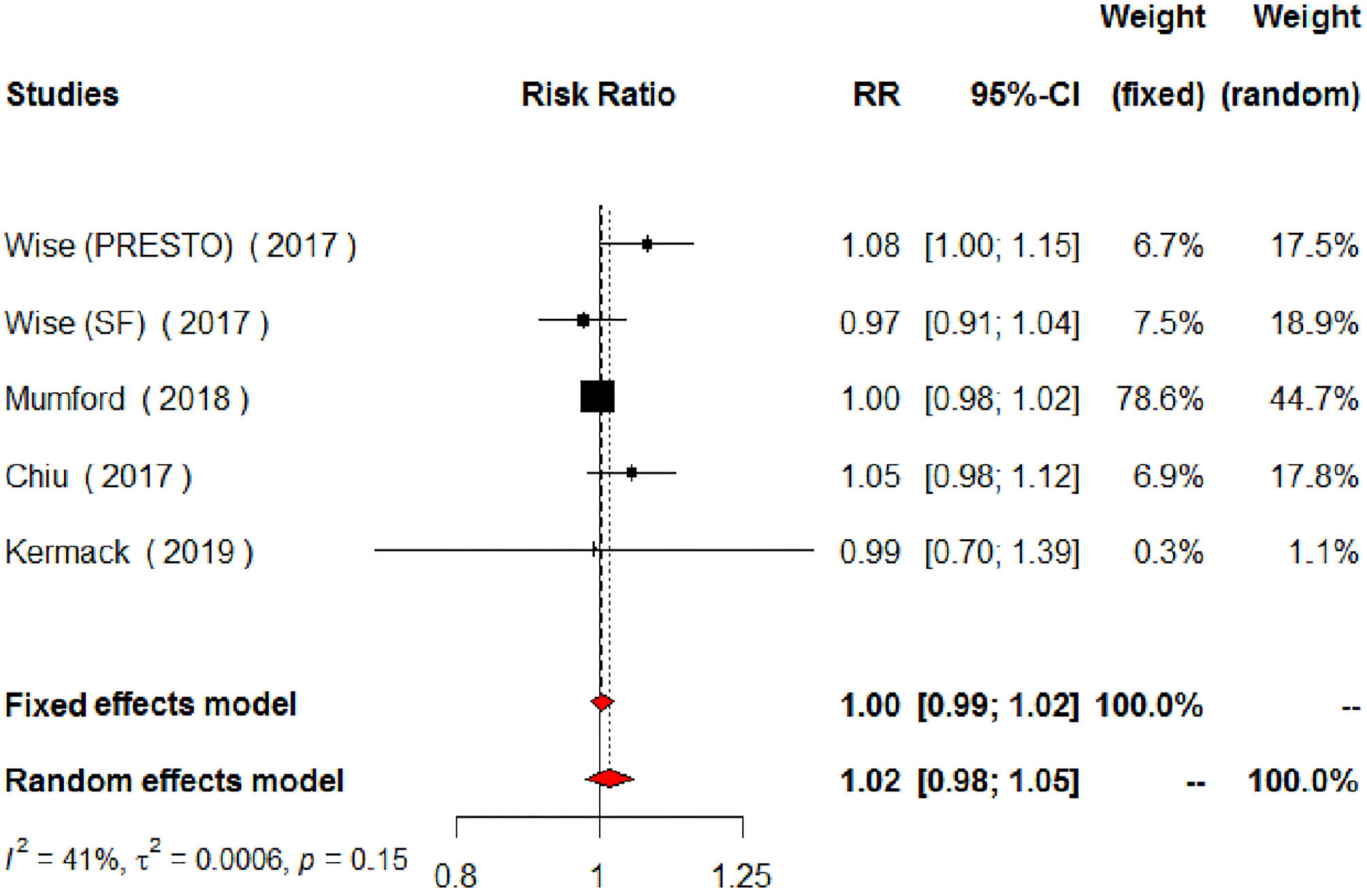
Forest plot for the meta-analysis of the relationship between periconceptional intake of ω-3 PUFAs and pregnancy achievement (*n* = 2,095).

**Figure 5:**
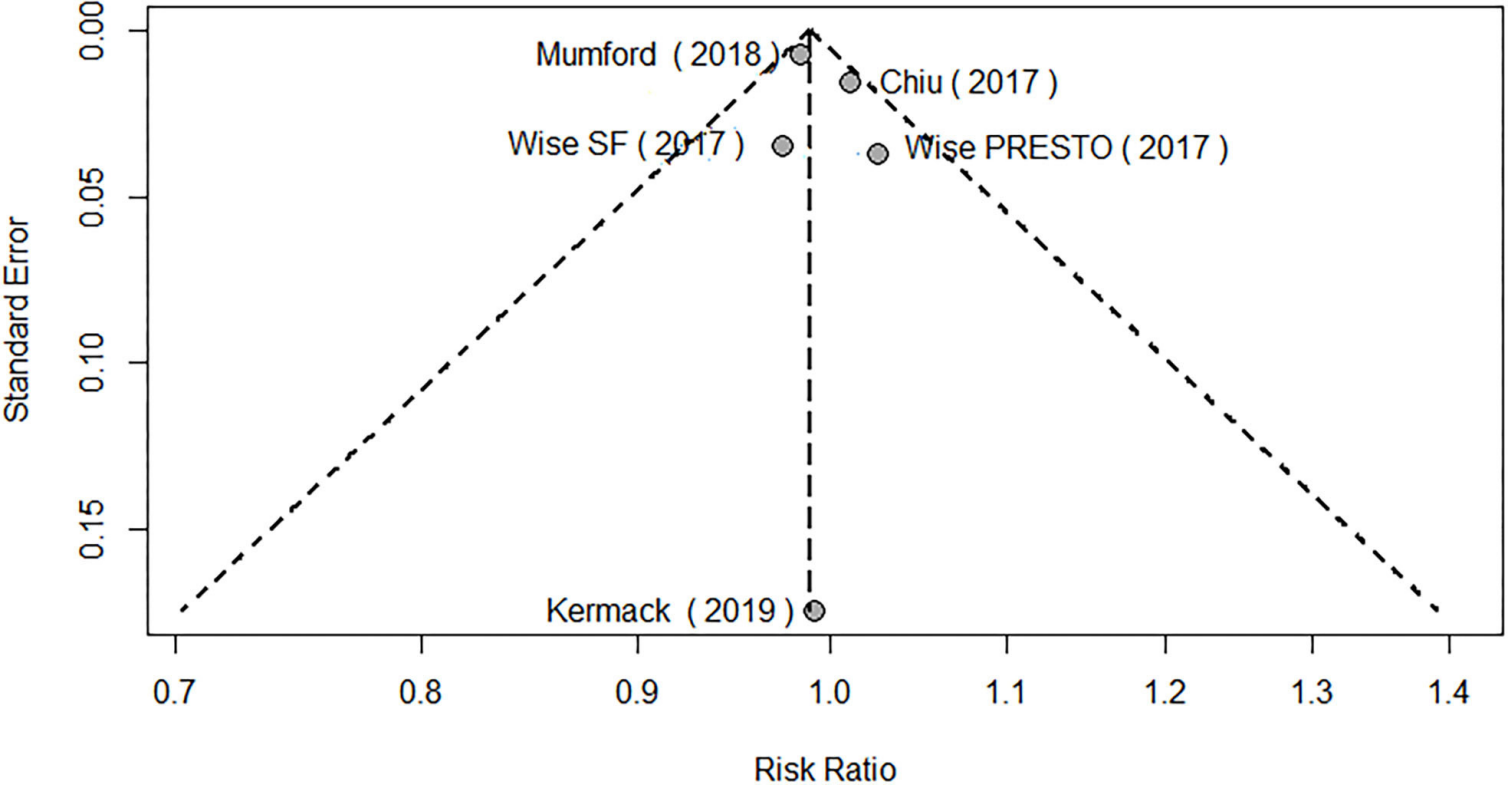
Funnel plot of the eligible PUFA studies.

The meta-analysis of the eligible four studies addressing periconceptional PUFA intake showed no significant effects on the achievement of pregnancy compared to controls (RR = 0.99, 95% CI 0.98–1.00, *p* = 0.0768) according to both the fixed effects model and the random effects model (Figure 2). As a secondary analysis, we next separately addressed ω-6 PUFAs and ω-3 PUFAs. After excluding one study, we analyzed ω-6 PUFA intake and the meta-analysis of the three eligible studies (one of them showing results of two separate populations) showed no significant effects on achieving pregnancy (RR = 0.99, 95% CI 0.98–1.01, *p* = 0.3210) compared to controls (Figure 3). Similarly, when we analyzed ω-3 PUFA intake, the meta-analysis of the four studies (one of them addressing two separate populations) showed no significant effects on achieving pregnancy according to the fixed effects model (RR = 1.00, 95% CI 0.99–1.02, *p* = 0.6809) and the random effects model (RR = 1.02, 95% CI 0.98–1.05) compared to controls (Figure 4). No heterogeneity was found among studies evaluating total PUFAs (*I*^2^ = 0%, *p* = 0.4370) and ω-6 PUFAs (*I*^2^ = 0%, *p* = 0.71, *p* = 0.4075), and low heterogeneity was found in studies evaluating ω-3 PUFAs (*I*^2^ = 41%, *p* = 0.1491). No bias due to small-study effects was found among these studies as visually assessed through a funnel plot (Figure 5).

### MUFAs and Pregnancy

**Figure 6:**
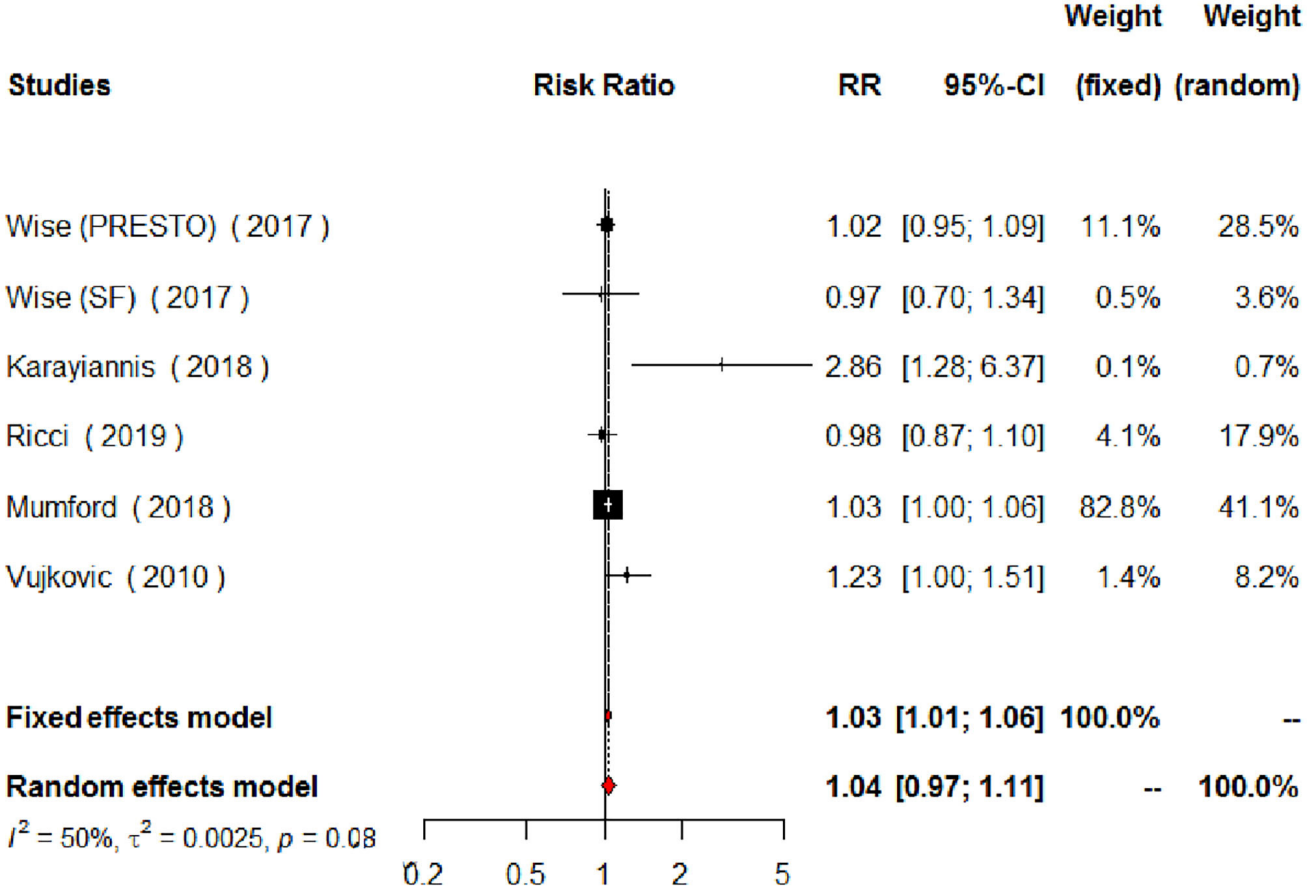
Forest plot for the meta-analysis of the relationship between periconceptional intake of MUFAs and pregnancy achievement (*n* = 2,473).

**Figure 7:**
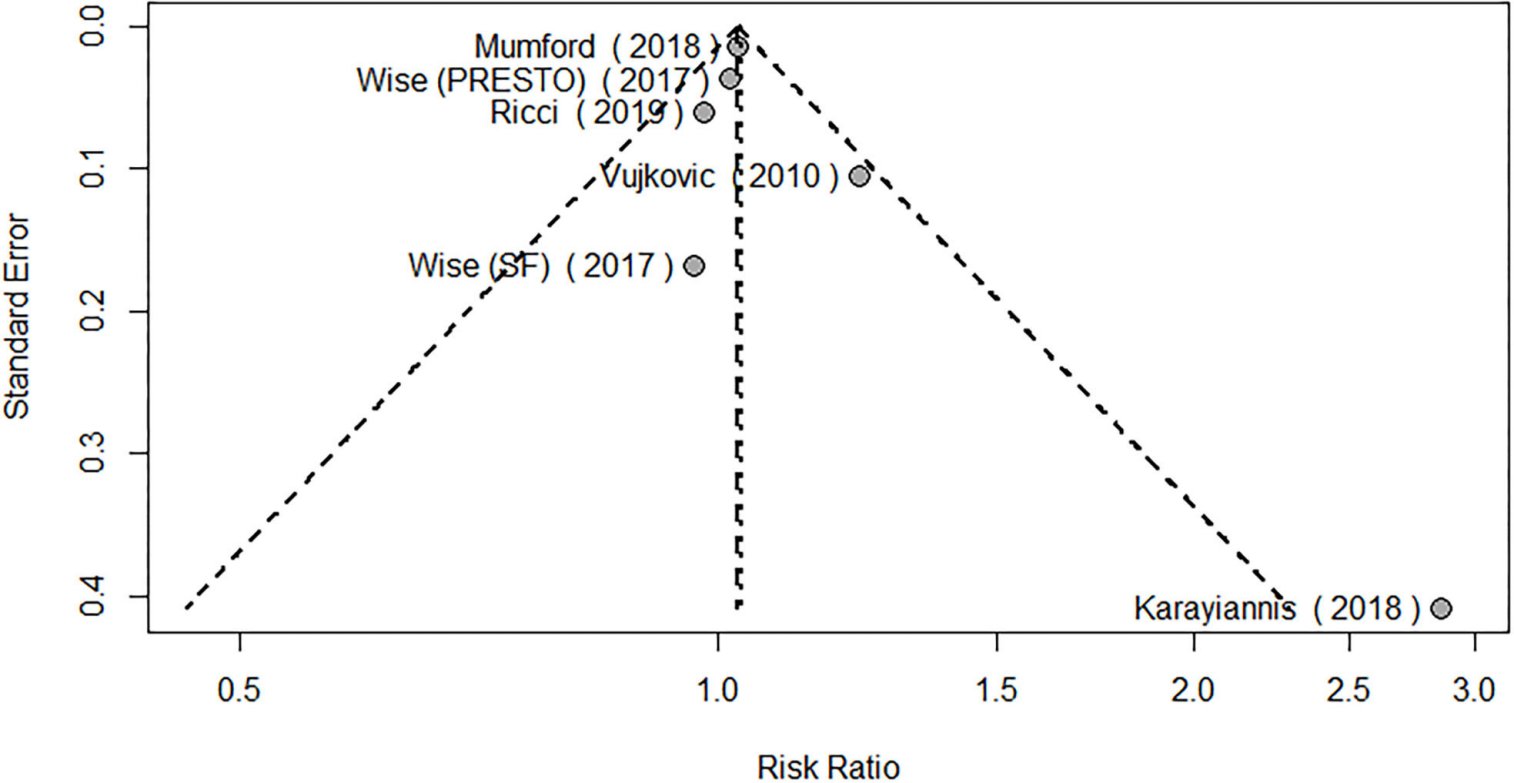
Funnel plot of the eligible MUFA studies.

**Figure 8:**
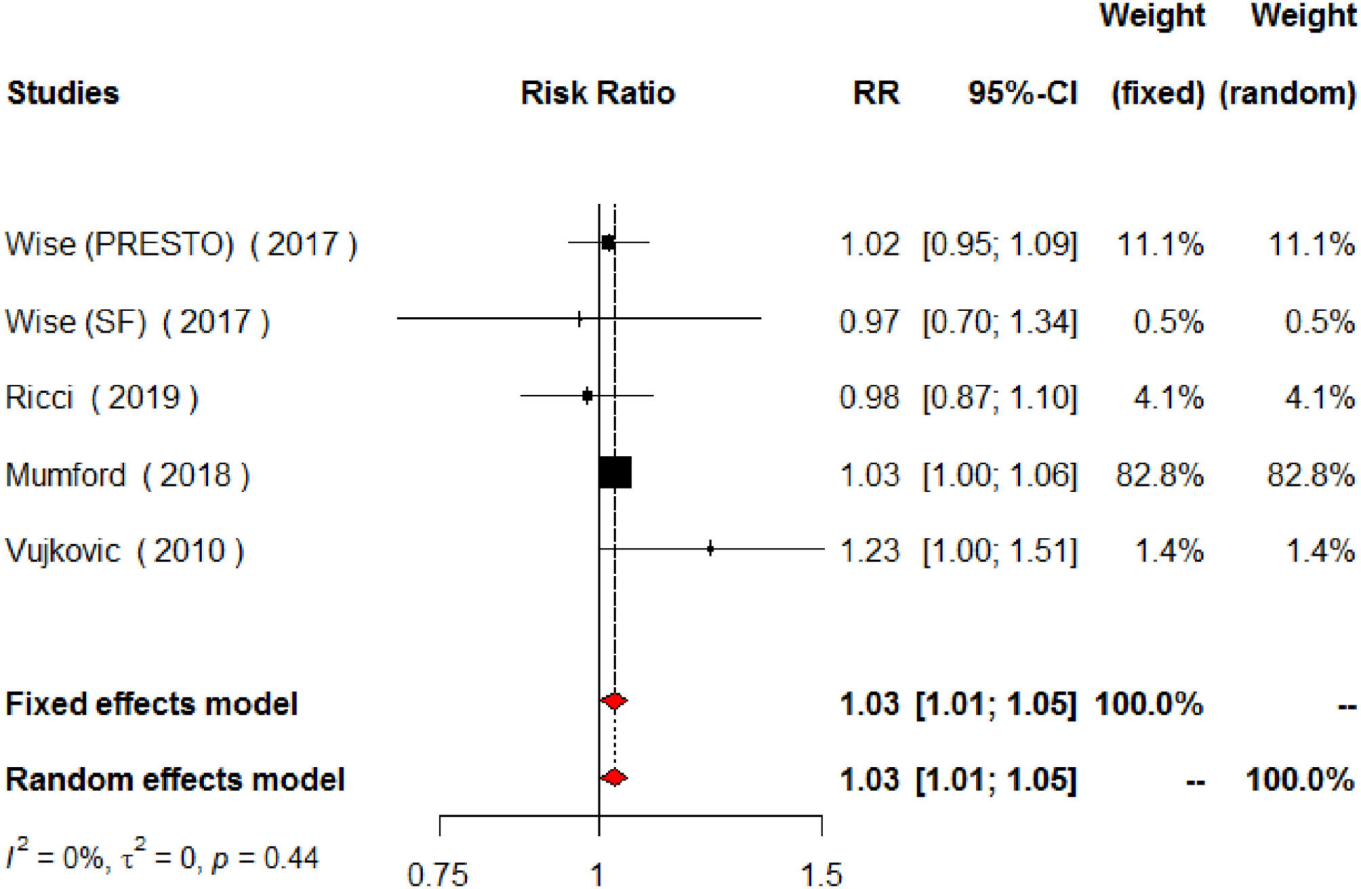
Forest plot for the secondary meta-analysis of the relationship between periconceptional intake of MUFAs and pregnancy achievement (*n* = 2,308).

Regarding MUFAs, the meta-analysis of the five studies addressing periconceptional dietary intake of MUFAs (one of them addressing two separate populations) showed a significant effect on achieving pregnancy when compared to controls according to the fixed effects model (RR = 1.03, 95% CI 1.01–1.06, *p* = 0.0146), although not significant effects according to the random effects model (RR = 1.04, 95% CI 0.97–1.11, *p* = 0.2713) (Figure 6). Moderate heterogeneity was found among the eligible studies evaluating MUFAs (*I*^2^ = 50%, *p* = 0.0764). The forest plot analysis and funnel plot visual assessment show that heterogeneity and small-study effects were mainly due to one study (Figures 6, 7). Therefore, we performed a secondary meta-analysis excluding this study. The evaluation of the relation between MUFA intake and pregnancy in the four remaining studies (one of them showing results of two separate populations) showed significant effects on achieving pregnancy according to both the fixed effects model and the random effects model (RR = 1.03, 95% CI 1.01–1.05, *p* = 0.0180) (Figure 8). No heterogeneity was found among these studies evaluating MUFAs (*I*^2^ = 0%, *p* = 0.4433).

## Discussion

Although several studies have suggested that periconceptional diets enriched in unsaturated fatty acids are able to promote fertility, reported data are still controversial (Gaskins and Chavarro, 2018; Lass and Belluzzi, 2019). The present meta-analysis suggests that unsaturated fatty acids induce moderate but positive effects on pregnancy achievement and that MUFAs are the unsaturated fatty acids that most contribute to the promotion of pregnancies. Of note, this is the first meta-analysis comparing evidence of the relationship between periconceptional intake of MUFAs and PUFAs with pregnancy achievement.

Experimental studies have clearly stated the relevant role of PUFAs during the periconceptional period and early gestation (Norwitz et al., 2001; Jawerbaum and Gonzalez, 2005; Wathes et al., 2007). Besides, both experimental models and human studies have shown associations between pathologies like obesity and diabetes (in which a prooxidant and proinflammatory environment is generated and pregnancy success is compromised) and reduced levels of PUFAs and impaired related signaling pathways (Lappas et al., 2011; Higa and Jawerbaum, 2013; Matorras et al., 2020).

Previous meta-analyses have addressed the role of ω-3 PUFAs in different gestational periods and gestational or perinatal diseases, finding controversial results. One of these studies has reported that ω-3 PUFA supplementation during pregnancy enhances pregnancy duration in low-risk patients but found no effects on neonatal growth (Szajewska et al., 2006). A meta-analysis evaluating the effect of ω-3 PUFA supplementation during pregnancy failed to find sufficient evidence to determine its benefit on perinatal depression (Suradom et al., 2021). On the other hand, the results of a meta-analysis have suggested that ω-3 PUFA intake may be beneficial to prevent childhood allergic diseases (Best et al., 2016).

In the present work, we evaluated the effect of MUFA, ω-3 PUFA and ω-6 PUFA intake on pregnancy success, because our previous basic studies showed that these bioactive lipid agents are capable of activating nutritional signals that prevent embryo loss even in a context of a prooxidant and proinflammatory intrauterine environment (Higa et al., 2010, 2014; Roberti et al., 2021). Both MUFAs and PUFAs, including essential PUFAs, have relevant biological functions such as nutrigenomic functions, epigenetic functions, activation of the nuclear receptors PPARs, activation of free fatty acid receptors (FFAR), regulation of the fluidity and function of biological membranes and/or regulation of prooxidant/proinflammatory pathways (Jawerbaum and Capobianco, 2011; Li et al., 2019; Kimura et al., 2020; Bordoni et al., 2021). In each tissue, and according to the physiological process (including ovulation, decidualization, implantation, placental development, embryo development), the biological function and formation of each PUFA derivative will rely, among others, on the levels of essential precursors, the enzymatic activity of delta desaturases, and different epigenetic factors (Jawerbaum and Gonzalez, 2005; Jawerbaum and Capobianco, 2011; Czumaj and Sledzinski, 2020). Besides, the antioxidant status clearly plays a role in PUFAs metabolization (Blokhina et al., 2003). In rat studies, we have previously found that a diet enriched in extra virgin olive oil, which is highly enriched in oleic acid (18:1 ω-9, ~70%) but also contains linolenic acid (18:2 ω-6, ~15%) and ALA (18:3 ω-3, ~0.5%), increases both ω-6 and ω-3 PUFAs in the fetal lungs (Kurtz et al., 2014), while diets enriched in either essential ω-6 or ω-3 PUFAs induce beneficial changes in the embryo, placenta and fetal organs from diabetic dams (Higa et al., 2010; Jawerbaum and Capobianco, 2011; Capobianco et al., 2018; Roberti et al., 2021). This suggests that the supplementation of essential PUFAs exerts relevant biological functions in reproductive processes, although the mechanisms involved are complex and may include their function as PPAR activators, FFAR activators, or membrane cell function regulators, among others, exerted in part as a result of their highly active derivatives from the ω-6 or ω-3 PUFAs series (Jawerbaum and Gonzalez, 2005; Jawerbaum and Capobianco, 2011; Bordoni et al., 2021). Considering this, the present meta-analysis addressed the putative relevance of unsaturated fatty acids as nutrigenomic agents that may lead to the proper activation of biological signaling pathways relevant in reproduction.

In this meta-analysis, the number of studies eligible was limited and allowed the evaluation of a total of 2,708 patients. Regarding the effects of PUFAs on achieving pregnancies, the eligible studies allowed the evaluation of a total of 2,121 patients. Considering these studies, we found no significant effect of PUFAs on pregnancy achievement. Regarding the effects of MUFAs on achieving pregnancies, the number of eligible studies allowed the evaluation of a total of 2,473 patients. Interestingly, considering all the eligible studies in the fixed effects model, as well as excluding one small-number study and using both fixed and random effects models, we found that a periconceptional diet enriched in MUFAs can promote pregnancy by 3–4%.

This study shows several limitations: (i) the number of eligible studies was low; (ii) the age of the women evaluated ranged between 18 and 45 years old; (iii) the eligible studies included different sources, dosages and means of intake estimation of dietary unsaturated fatty acids; (iv) some studies included obese patients whereas others did not; (v) some studies evaluated patients undergoing assisted reproduction whereas others did not; and (vi) although adjusted effect estimates were used, some degree of residual confounding bias at study level could remain. Of note, there were two studies in which the intake of unsaturated fatty acids was estimated by their high plasma levels, highly associated with a high intake of unsaturated fatty acids (Calder, 2018). Interestingly, despite the described limitations, heterogeneity in the PUFAs studies was low. Differently, heterogeneity in the MUFAs studies was moderate, due to a study showing a marked positive effect on clinical pregnancies (185% increase in the RR) but evaluating a low number of patients (Karayiannis et al., 2018). The lack of heterogeneity due to the different populations assessed and type of unsaturated fatty acid supplementation suggests that different forms of unsaturated fatty acid supplementation may be used and that different populations may benefit from an increased intake of unsaturated fatty acids.

To address whether the lack of effect of PUFA intake on pregnancy success was due to the type of PUFA evaluated, we performed a secondary meta-analysis addressing the differential effects of ω-6 and ω-3 PUFAs on pregnancy achievement. Results showed that neither an increased intake of ω-6 PUFAs nor an increased intake of ω-3 PUFAs promotes pregnancy achievement. This result was surprising considering the relevant role of dietary PUFAs and the specific functions of individual PUFAs of the ω-6 and ω-3 series: arachidonic acid derivatives, DHA and EPA, which are differentially generated from their precursors and relevant in ovulation, decidualization, implantation and embryo organogenesis (Norwitz et al., 2001; Jawerbaum and Gonzalez, 2005). Indeed, the balance between proinflammatory and antioxidant effects is regulated by ω-6 and ω-3 PUFAs, and the antioxidant effects are the result, at least in part, of the activation of PPAR receptors and FFAR receptors, and the production of resolvins or 15deoxydelta^12, 14^PGJ_2_, derivatives of PUFAs of the ω-3 and ω-6series, respectively (Scher and Pillinger, 2005; Jawerbaum and Capobianco, 2011; Calder, 2017; Kimura et al., 2020).

On the other hand, several studies have shown the susceptibility of PUFAs to degradation and the capacity to form toxic components which may prevent a beneficial effect (Al-Gubory et al., 2010). Also, when ω-3 PUFAs are ingested as fish or fish-derived supplements, there are fish contaminants that may counteract the beneficial effects of PUFAs (Buck et al., 2000; Langer et al., 2007). Of note, in the present meta-analysis, we were not able to include a study showing the ability of ω-3 PUFAs to improve embryo morphology, as it did not report data on clinical/biochemical diagnosis of pregnancy (Hammiche et al., 2011). Also, a study showing the effect of increased ω-6/ω-3 PUFAs ratio in pregnancy success was excluded as it reported only the ratio but not the intake of these PUFAs (Jungheim et al., 2013). Interestingly, increased DHA and EPA were found in the follicular fluid of women receiving a diet supplemented with marine ω-3 PUFAs, suggesting an influence of dietary supplementation on the generation of PUFAs of relevant biological activity during the process of ovulation (Kermack et al., 2021). In addition, in a recent meta-analysis, ω-3 PUFA supplementation was found highly related to the improvement of oxidative stress status in women with gestational diabetes mellitus (GDM) (Chatzakis et al., 2021). As GDM is not a periconceptional disease, further studies addressing ω-3 PUFA supplementation in pathologies that promote increased oxidative stress in the periconceptional period, like obesity or pre-gestational diabetes, are needed.

On the other hand, in this study, we assessed the intake of MUFAs, which were included or not in a Mediterranean diet, and the low heterogeneity found suggests that different ways of MUFAs consumption are beneficial for pregnancy success. Out of pregnancy, dietary MUFAs promote healthy blood lipid profiles, mediate blood pressure regulation, improve insulin sensitivity and are protective against cardiovascular disease risk factors and metabolic syndrome (Gillingham et al., 2011). Oleic acid, the most common dietary MUFA, is the main unsaturated fatty acid in oocytes (Matorras et al., 1998). Oleic acid has been shown to counteract detrimental effects of saturated fatty acids during oocyte development and to contribute to pre-implantation embryo development through mechanisms which likely involve membrane structural organization, attenuation of oxidative stress and regulation of intracellular signaling (Fayezi et al., 2018). Further studies addressing the mechanisms involved in the beneficial effects provided by increased MUFA consumption in pregnancy achievement are needed.

Although the fetus is not a direct subject of the present study, it is important to point out that both ω-6 PUFAs and ω-3 PUFAs play a crucial role in influencing fetal brain development (Innis, 2005). Therefore, together with the benefits of a periconceptional diet enriched in MUFAs, a diet that provides sufficient ω-6 and ω-3 PUFAs is needed from the periconceptional stage and throughout pregnancy (Hanson et al., 2015; Comerford et al., 2016). In addition, the relevance of the intake of MUFAs and PUFAs extends to the lactation period, in which it is directly related to the quality of the milk (Comerford et al., 2016). As a low MUFAs and PUFAs intake and circulating levels have been found in pregnant women, the evaluation and improvement of this status are encouraged (Sioen et al., 2017; Hoge et al., 2018).

In conclusion, this meta-analysis suggests that the intake of MUFAs, although not that of PUFAs, in the periconceptional period could increase the pregnancy rate in women attending or not assisted reproduction programs. This suggests that pregnancy success may be improved through periconception diets enriched in MUFAs and highlights the relevance of nutritional interventions to improve women's fertility.

## Data Availability Statement

The raw data supporting the conclusions of this article will be made available by the authors, without undue reservation.

## Author Contributions

AJ and JM designed the study. CG and DG extracted data and performed data analysis. AJ, JM, CG, and DG contributed to the literature search, interpretation, and writing and proofreading of the manuscript. All authors contributed to the article and approved the submitted version.

## Funding

This work was supported by the Agencia Nacional de Promoción Científica y Tecnológica de Argentina (PICT 2017-126 and PIDC 2015-064).

## Conflict of Interest

The authors declare that the research was conducted in the absence of any commercial or financial relationships that could be construed as a potential conflict of interest.

## Publisher's Note

All claims expressed in this article are solely those of the authors and do not necessarily represent those of their affiliated organizations, or those of the publisher, the editors and the reviewers. Any product that may be evaluated in this article, or claim that may be made by its manufacturer, is not guaranteed or endorsed by the publisher.
